# Electrode Position and Current Amplitude Modulate Impulsivity after Subthalamic Stimulation in Parkinsons Disease—A Computational Study

**DOI:** 10.3389/fphys.2016.00585

**Published:** 2016-11-29

**Authors:** Alekhya Mandali, V. Srinivasa Chakravarthy, Roopa Rajan, Sankara Sarma, Asha Kishore

**Affiliations:** ^1^Department of Biotechnology, Bhupat and Jyoti Mehta School of Biosciences, Indian Institute of Technology MadrasChennai, India; ^2^Department of Neurology, Comprehensive Care Centre for Movement DisordersTrivandrum, India; ^3^Achutha Menon Centre for Health Science Studies, Sree Chitra Tirunal Institute for Medical Sciences and TechnologyTrivandrum, India

**Keywords:** impulsivity, sub thalamic stimulation, Parkinson's disease, Iowa gambling task, reinforcement learning

## Abstract

**Background:** Subthalamic Nucleus Deep Brain Stimulation (STN-DBS) is highly effective in alleviating motor symptoms of Parkinson's disease (PD) which are not optimally controlled by dopamine replacement therapy. Clinical studies and reports suggest that STN-DBS may result in increased impulsivity and *de novo* impulse control disorders (ICD).

**Objective/Hypothesis:** We aimed to compare performance on a decision making task, the Iowa Gambling Task (IGT), in healthy conditions (HC), untreated and medically-treated PD conditions with and without STN stimulation. We hypothesized that the position of electrode and stimulation current modulate impulsivity after STN-DBS.

**Methods:** We built a computational spiking network model of basal ganglia (BG) and compared the model's STN output with STN activity in PD. Reinforcement learning methodology was applied to simulate IGT performance under various conditions of dopaminergic and STN stimulation where IGT total and bin scores were compared among various conditions.

**Results:** The computational model reproduced neural activity observed in normal and PD conditions. Untreated and medically-treated PD conditions had lower total IGT scores (higher impulsivity) compared to HC (*P* < 0.0001). The electrode position that happens to selectively stimulate the part of the STN corresponding to an advantageous panel on IGT resulted in de-selection of that panel and worsening of performance (*P* < 0.0001). Supratherapeutic stimulation amplitudes also worsened IGT performance (*P* < 0.001).

**Conclusion(s):** In our computational model, STN stimulation led to impulsive decision making in IGT in PD condition. Electrode position and stimulation current influenced impulsivity which may explain the variable effects of STN-DBS reported in patients.

## Introduction

Deep brain stimulation (DBS) of the subthalamic nucleus (STN), is a surgical technique now widely applied for the treatment of Parkinson's disease (PD) when dopamine replacement therapy fails to provide sustained relief of motor symptoms or induces drug-induced dyskinesias (Benabid, 2003). Though the exact mechanism of action of DBS is not well-established, it is known that stimulation disrupts (Rosa et al., 2012) the increased synchrony and bursting activity in the β band (8–30 Hz; Kühn et al., 2006) of the STN neurons.

Several reports have highlighted the development of new onset, often transient, impulse control disorders (ICDs) following STN stimulation (Hershey et al., 2004; Smeding et al., 2007; Combs et al., 2015). This was thought to be, due to stimulation of the cognitive sub territory of STN or the spread of stimulation to adjacent parts of the cortico-limbic circuits. In support of this theory, stimulation parameters such as current spread and electrode position were shown to affect the outcome in cognitive tasks in PD patients (Sudhyadhom et al., 2007; York et al., 2009; Witt et al., 2013). Stimulation of the ventral STN decreased the number of correct hits and increased the number of errors on commission in Go-No Go task, when compared to stimulation to dorsal STN (Hershey et al., 2010). STN stimulation can also increase risk taking behavior in Iowa Gambling Task (IGT; Evens et al., 2015). Patients with STN-DBS tended to overestimate their performance with a preference toward competitive environments (Florin et al., 2013). On the other hand, pre-existing ICDs were reported to resolve following STN-DBS, as a result of reduction in dopaminergic medication (Castrioto et al., 2015). Thus STN-DBS may lead to varying net effects on impulsivity in PD through different mechanisms. Clinical studies aimed at dissecting out the effect of STN stimulation on impulsivity have been limited by the confounding effects of therapeutic reduction of dopaminergic medication following STN-DBS.

Computational models provide an opportunity to overcome this limitation by simulating the effect of variations in stimulation and medication protocols individually which cannot be easily applied in human subjects. We hypothesized that electrode position and stimulation parameters affect the decision making ability of PD patients who received STN-DBS. We used a spiking network model of basal ganglia (BG; Mandali et al., 2015) to test the performance under various conditions on the standard gambling task, IGT (Evens et al., 2015). It is known that IGT captures certain impulsive features such as the risk taking ability (Fukui et al., 2005) and lack of premeditation (Zermatten et al., 2005) during decision making. PD patients are known to have poor IGT performance, especially during dopaminergic medication (Poletti et al., 2011; Gescheidt et al., 2012).

Simulating IGT requires learning, which was incorporated in the proposed model using Reinforcement learning (RL; Chakravarthy et al., 2010). RL describes the manner in which an agent learns stimulus-response (S-R) relations based on action outcomes: S-R pairs associated with rewarding outcomes are reinforced while those associated with punishment are attenuated (Dayan and Abbott, 2001). Experimental evidence shows that dopamine (DA) codes for reward prediction error or the temporal difference error term (“δ”) in RL (Niv, 2009).

Using the spiking network model of BG (described in Section Materials and Methods), we studied the performance of the model in IGT in normal, PD with and without medication [L-DOPA and Dopamine Agonists (DAA)] and STN-DBS conditions. Our results show that model in normal condition was able to learn from bad choices during the initial trials and improved its performance as the trials progressed. This was observed to be absent in PD with medication (both L-DOPA and DAA) condition.

We then studied the effect of STN stimulation alone on learning and performance by comparing it PD with and without medication. The simulation results show that during the initial trials, the stimulation current interferes with the learning which is reflected as poor performance. We then proceeded to study the factors of stimulation such as electrode position, amplitude of the current and spread that are specific to a patient. The simulation results indicate that electrode position played a significant role in altering performance in the model. We also observed that the model's performance improved for a narrow band of current amplitude.

## Materials and methods

### Spiking neuron model of the basal ganglia

**Figure 1:**
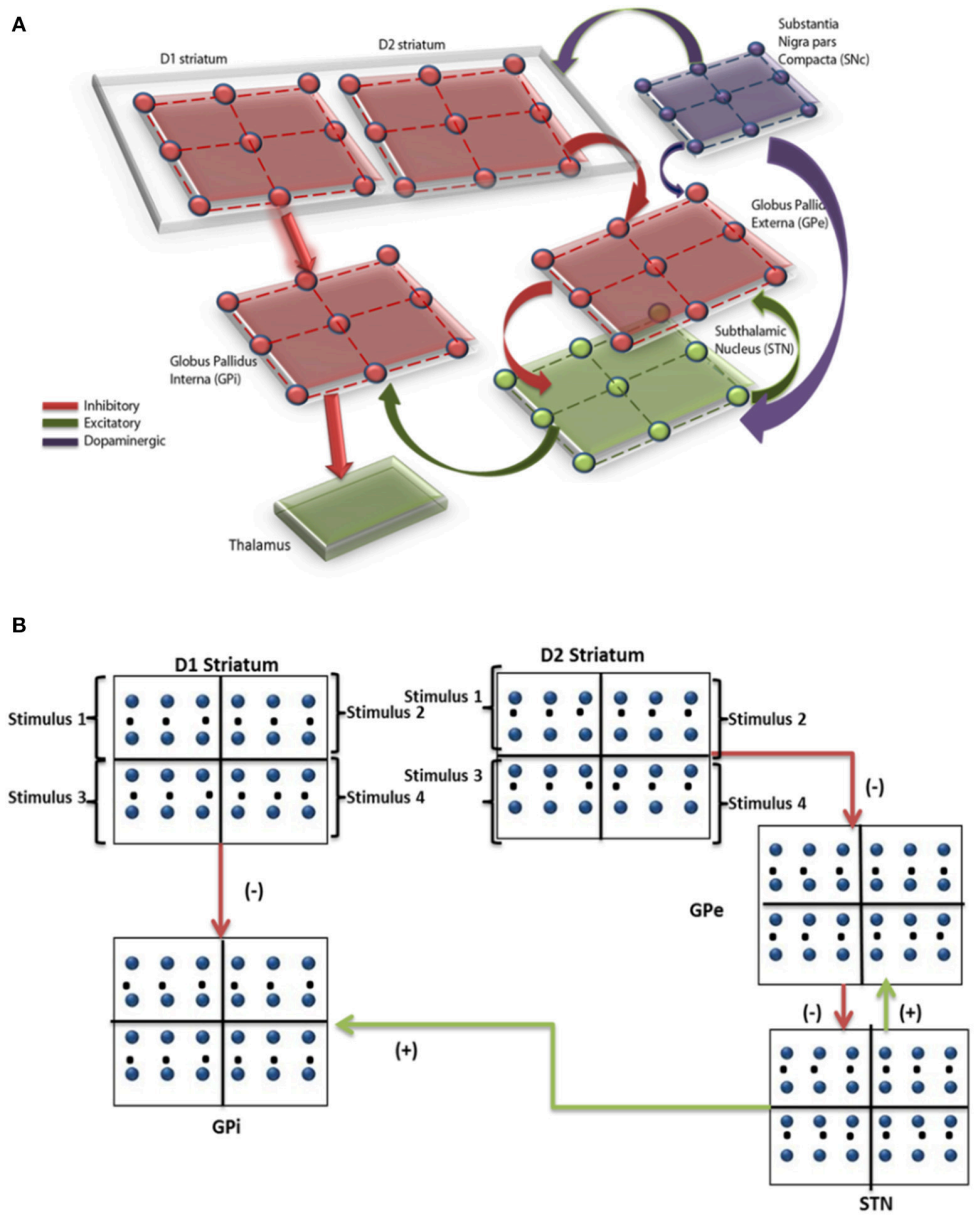
**(A)** Shows the computational spiking basal ganglia model with key nuclei such as striatum (D1, D2), STN, GPe, GPi, and thalamus. Excitatory/inhibitory/modulatory glutamatergic/GABAergic/dopaminergic projections are shown by green/red/violet arrows. **(B)** Shows the BG model and the regions within each nuclei corresponding to the 4 decks are indicated.

The network model of BG (Mandali et al., 2015; Figure 1A) was built using 2-variable Izhikevich spiking neurons (Izhikevich, 2003) where each nucleus was modeled as a 2D array (=50 × 50). Parameters for each of the nuclei [STN, Globus Pallidus internus (GPi) and externus (GPe) were chosen such that the model neurons display firing patterns (in terms of firing rate and firing patterns such as rebound firing) of their biological counterparts (Mandali et al., 2015). STN and GPe neurons were bi-directionally connected (Plenz and Kital, 1999) where projections from GPe (STN) are inhibitory (excitatory). Each GPi neuron received both glutamatergic projections from STN and GABAergic projections from D1R-expressing medium spiny neurons (MSNs) of the striatum (Gerfen and Surmeier, 2011). The final action selection was done at thalamus which was simulated as a race model (Vickers, 1970). The activities of both D1- and D2-receptor expressing, striatal MSNs that receive input from cortex (Tritsch and Sabatini, 2012) were modeled as Poisson spike trains. The full set of equations and module sizes related to the model are described in Appendix A and Table A.I (Datasheet in Supplementary Material). The input from cortex to STN, also known as hyper-direct pathway and the GABAergic projection from GPe to GPi, were not included in the model as their functional significance has not been fully explored. The list of acronyms and parameters used in the model are listed in Table 1 and Tables A.II, IV (Supplementary).

(1) $$\begin{array}{r} \frac{dv_{ij}^{x}}{dt}=0.04{\left(v_{ij}^{x}\right)}^{2}+5v_{ij}^{x}-u_{ij}^{x}+140+I_{ij}^{x}+I_{ij}^{syn} \end{array}$$

(2) $$\begin{array}{r} \frac{du_{ij}^{x}}{dt}=a\left(bv_{ij}^{x}-u_{ij}^{x}\right) \end{array}$$

(3) $$\begin{array}{l} if\;v_{ij}^{x}\geq v_{peak}\left\{\begin{array}{l} v_{ij}^{x}\leftarrow c\\ u_{ij}^{x}\leftarrow u_{ij}^{x}+d \end{array}\right\} \end{array}$$

where, $v_{ij}^{x}$ = membrane potential, $u_{ij}^{x}$ = membrane recovery variable, $I_{ij}^{Syn}$ = total synaptic current received, $I_{ij}^{x}$ = external current applied to neuron “*x*” at location (*i, j*), *v*_*peak*_ = maximum voltage (+30 mv) with *x* being STN/GPe/GPi neuron.

**Table 1:** **Lists the Acronyms and parameters used in this article**.

**Variable/Acronym**	**Full form**
STN	Sub Thalamic Nucleus
PD	Parkinson's Disease
DBS	Deep Brain Stimulation
IGT	Iowa Gambling Task
BG	Basal Ganglia
RL	Reinforcement Learning
DA	Dopamine
L-DOPA	Leva Dopa
DAA	Dopamine Agonist
GPe	Globus Pallidus externus
GPi	Globus Pallidus internus
Untreated PD/PD OFF	PD condition without medication
Medically treated PD/PD ON	PD condition with medication
HC	Healthy controls
MPTP	1-methyl-4-phenyl-1,2,3,6-tetrahydropyridine
ICD	Impulse Control disorder
GABA	Γ-Aminobutyric acid
δ	Error term similar to dopamine
σ	Parameter that controls the spread of the current
$w_{i,k}^{D1}$	Cortico-striatal weight to D1 striatum
$w_{i,k}^{D2}$	Cortico-striatal weight to D2 striatum
A_*DBS*_	Parameter that controls the amplitude of the DBS current
δ_*lim*_	Clamped DA-value resembling PD condition
δ_*med*_	DA medication
η	(=0.1) Learning rate of the model
R^sync^	Synchronization measure

### Behavioral task-IGT

The task involved presentation of four decks of cards wherein each of the decks A/B/C/D is associated with a combination of reward and penalty. IGT was conducted for a total of 100 trials (5 bins of 20 trials each).The net outcome of a certain card selection (reward + penalty) in each trial was calculated. The probability and amount of penalty varied from deck to deck as explained in Table A.III (Datasheet in Supplementary Material). Over a few trials, one can observe that cards from the decks A and B (C and D) were disadvantageous (advantageous) as the corresponding expected value is negative (positive) The performance was measured as IGT total score (number of selections from “C,” “D”—number of selections from “A,” “B”)

### Simulating IGT using spiking neuron network model

Since IGT consists of 4 decks, each nucleus [STN/GPe/GPi/ Striatum (both D1 and D2)] in the network was divided equally into 4 quadrants, where each quadrant received input from one of the decks (Figure 1B).The expected value of each card was represented by the cortico-striatal weight which was modulated by DA term “δ.” The input to GPe and GPi (i.e., the output of D2 and D1 striatum) was modeled as Poisson spike train (Reti, 2015), whose frequency was proportional to the cortico-striatal weight ($w_{i,k}^{D1}$, $w_{i,k}^{D2}$) of the corresponding card (*i*) and trial (*k*). The striatal neuronal firing rate was restricted to 2–40 Hz as per experimental literature (Kravitz et al., 2010). Since DA is known to modulate plasticity in cortico-striatal conditions, the error term “δ” (in the model) was used to update the cortico-striatal synapses (Surmeier et al., 2007). DA also modulated the synaptic strengths within various BG nuclei such as STN (Cragg et al., 2004), GPe (Smith and Kieval, 2000).

#### Cortico striatal weight update and temporal difference error

Each deck was associated with 2 cortico-striatal weights ($w_{i,0}^{D1}$, $w_{i,0}^{D2}$) which were initialized with random values from a uniform distribution over (0, 1). The two cortico-striatal weights were trained as,

(4) $$\begin{array}{r} \Delta w_{i,k+1}^{D1}=\eta \delta_{k}x_{i,k}^{inp} \end{array}$$

(5) $$\begin{array}{r} \Delta w_{i,k+1}^{D2}=-\eta \delta_{k}x_{i,k}^{inp} \end{array}$$

The expected value (*V*_*k*_) for *k*th trial was calculated as,

(6) $$\begin{array}{l} V_{\text{k}}=\overset{4}{\underset{i=1}{\sum }}w_{i,k}^{D1}*x_{i,k}^{sel} \end{array}$$

The reward (Re_*k*_) for *k*th trial was calculated as,

(7) $$\begin{array}{l} \text{R}{\text{e}}_{\text{k}}=\overset{4}{\underset{i=1}{\sum }}r_{i,k}*x_{i,k}^{sel} \end{array}$$

The loss (*L*_*k*_) for the *k*th trial was calculated as,

(8) $$\begin{array}{l} L_{\text{k}}=\overset{4}{\underset{i=1}{\sum }}l_{i,k}*x_{i,k}^{sel} \end{array}$$

The error (δ) for *k*th trial was defined as,

(9) $$\begin{array}{l} \delta_{k}={\text{Re}}_{k}+{\text{L}}_{k}-{\text{V}}_{k} \end{array}$$

where, $w_{i,k+1}^{D1}\left(w_{i,k+1}^{D2}\right),w_{i,k}^{D1}\left(w_{i,k}^{D2}\right)$ were the cortico-striatal weights of *D1* (*D2*) striatum for *i*th card in k+1th and kth trial, *r*_*i, k*_ and *l*_*i, k*_ were the reward and loss obtained for the selected ith card in kth trial, *x*^*inp*^ was the input binary vector representing the 4 decks, *x*^*sel*^ was the binary vector representing the selected card e.g., if the card “A” is selected *x*^*sel*^ = [1 0 0 0].

#### Simulating untreated PD and medically treated PD conditions

Bearing in mind that “δ” is similar to DA activity (Schultz, 1998; Niv, 2009) and there is loss of DA neurons in PD, we simulated PD condition by clamping the “δ” value (Equation 9) to a low limit (δ_*lim*_) which resembles the untreated PD condition (Equation 10).

(10) $$\begin{array}{r} \delta_{\lim}=\min\left(\delta ,DA_{ceil}\right) \end{array}$$

Where min (y,a) is defined as $\begin{array}{l} z=y\;if\;y<a\\ \;a\;if\;y>a \end{array}$ and *DA*_*ceil*_ is the upper limit of “δ.” Medically treated PD condition clinically involves external intake of dopamine precursors such as L-DOPA which was simulated by adding a positive “δ_*med*_” term to the δ_*lim*_ (Mandali and Chakravarthy, 2016) (Equation 10).

(11) $$\begin{array}{l} \delta_{new}=\delta_{\lim}+\delta_{med} \end{array}$$

Another class of medication prescribed to PD patients is DAA, which has differential affinity for dopamine receptors. We simulated DAA with preferential affinity for *D2* receptors, also known to be linked to impulsivity (MacMahon and Macphee, 2008). The δ_*new*_ in the Equation (11) was used to update only *D2* cortico-striatal weight (*w*^*D2*^) unlike for L-DOPA where both *w*^*D1*^ and *w*^*D2*^ were updated.

#### DBS current

An external current which mimics the clinically delivered DBS current was applied to the STN neurons in the model. The parameters (frequency, pulse duration, and amplitude) of the stimulation current were chosen to be similar to the typical values used in a clinical setting (Garcia et al., 2005) [Appendix A (Datasheet in Supplementary Material)]. The spread of current over network of neurons spatially is known to follow a Gaussian distribution (Lukasiewicz and Werblin, 1990). Apart from that, Foutz and McIntyre (2010) have simulated various stimulation waveforms and observed that non-rectangular waveforms are more efficient (Foutz and McIntyre, 2010). The stimulation current was applied to the entire/part of STN module in the form of Gaussian distribution (Foutz and McIntyre, 2010). The mean of the Gaussian coincides with the lattice position (*i*_*c*_, *j*_*c*_) which was assumed to be the center of the electrode and extent of current spread was controlled by the variance parameter (σ).

(12) $$\begin{array}{l} I_{ij}^{DBS}=A_{DBS}*e^{\frac{-\left({\left(i\;-\;i_{c}\right)}^{2}\;+\;{\left(j\;-\;j_{c}\right)}^{2}\right)}{\sigma^{2}}} \end{array}$$

where $I_{ij}^{DBS}$ is the current received by the neuron at position (*i, j*) added to equation 1 of STN neurons, *A*_*DBS*_ is the amplitude of the current (pA), σ controls the current spread and (*i*_*c*_, *jc*) is the mean/center point of the electrode. The effect of electrode position (*i*_*c*_, *j*_*c*_) and stimulation parameters *A*_*DBS*_ and σ on STN activity and on behavior was explored.

Values of the model parameters used for simulating various conditions (PD, medication and stimulation) are given in Appendix A (Datasheet in Supplementary Material).

### Statistical analysis

The IGT score obtained from each of the conditions [Healthy Controls (HC), untreated PD, medically treated PD, with and without STN stimulation] were compared using repeated measures of ANOVA. ANOVA (which stands for analysis of variance) is a popular statistical technique used to check if the means of two or more populations are equal and come from same distribution. It calculates the variance of means between the groups rather than intra group variance to determine whether the groups come from the same distribution.

Once the statistical test such as ANOVA has been performed, it is important to determine the underlying patterns in the data i.e., groups which could be representatives of different populations and the detects used are generally called as the a-posteriori tests. One such simple yet powerful test is the Bonferroni test which uses the “*p*-value” to determine the significance of the result. We have used the *post-hoc* Bonferroni test to study the effect of stimulation, medication on performance. All statistical tests were performed using IBM SPSS Statistics for Windows, Version 21.0, and Armonk, NY: IBM Corp., USA.

## Results

### De-synchronization by DBS current

**Figure 2:**
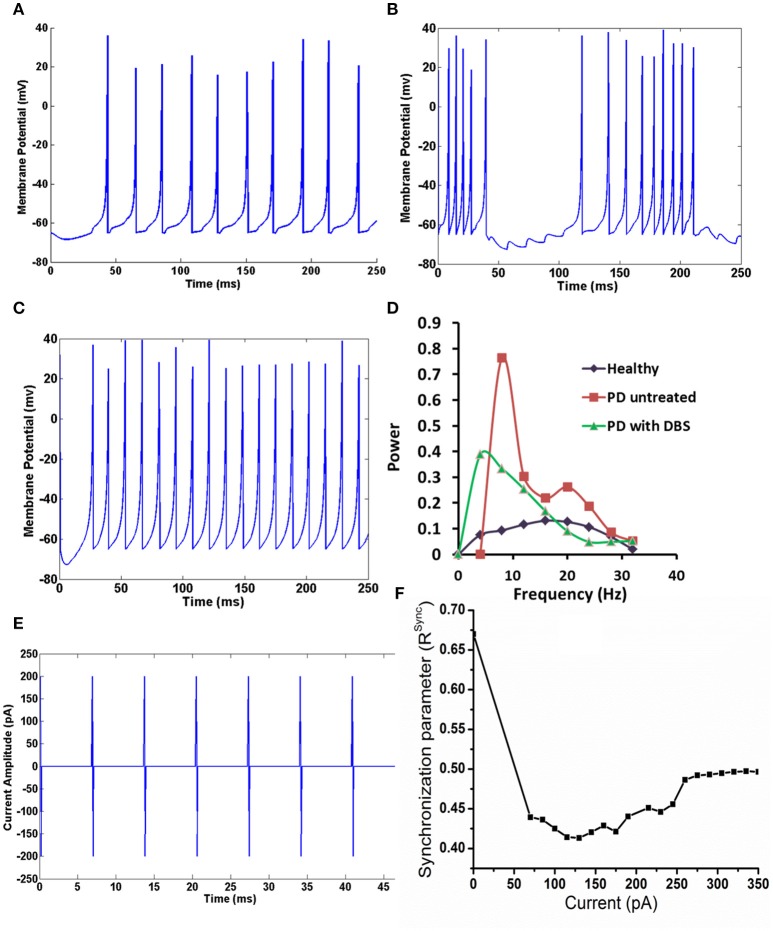
**The activity of STN neuron healthy, with and without DBS in PD. (A)** Shows the activity of STN neurons in healthy condition, **(B)** shows the bursting activity of STN neurons in PD condition. **(C)** STN neurons resume to tonic firing after DBS, **(D)** the reduction in the frequency content at tremor frequency (4 Hz) in STN neurons in mentioned conditions, **(E)** shows the DBS current in biphasic mode (frequency = 130 Hz with amplitude of 200 pA), **(F)** shows the synchronization levels in STN neurons with increase in DBS current.

The membrane potential of STN neurons PD untreated condition (Figures 2B,D) showed bursting activity and frequency content showed a peak at around 4 Hz with high synchrony level (=0.67; Mandali et al., 2015) which was absent in healthy condition (Figures 2A,D). On stimulating the STN neurons in PD condition, the peak around tremor frequency (=4 Hz) was significantly reduced (Figure 2D; *P* < 0.00001). Similarly the bursting activity in Figure 2B was overridden and suppressed by the stimulating current (Figure 2C). The synchrony level, *R*^sync^ (Equation A.13) in the presence of DBS current (Figure 2E) decreased from 0.67 (in PD condition and stimulation-OFF) to 0.42 (stimulation-ON; Figure 2F) but increased at higher current amplitudes.

### IGT-healthy vs. PD condition

**Figure 3:**
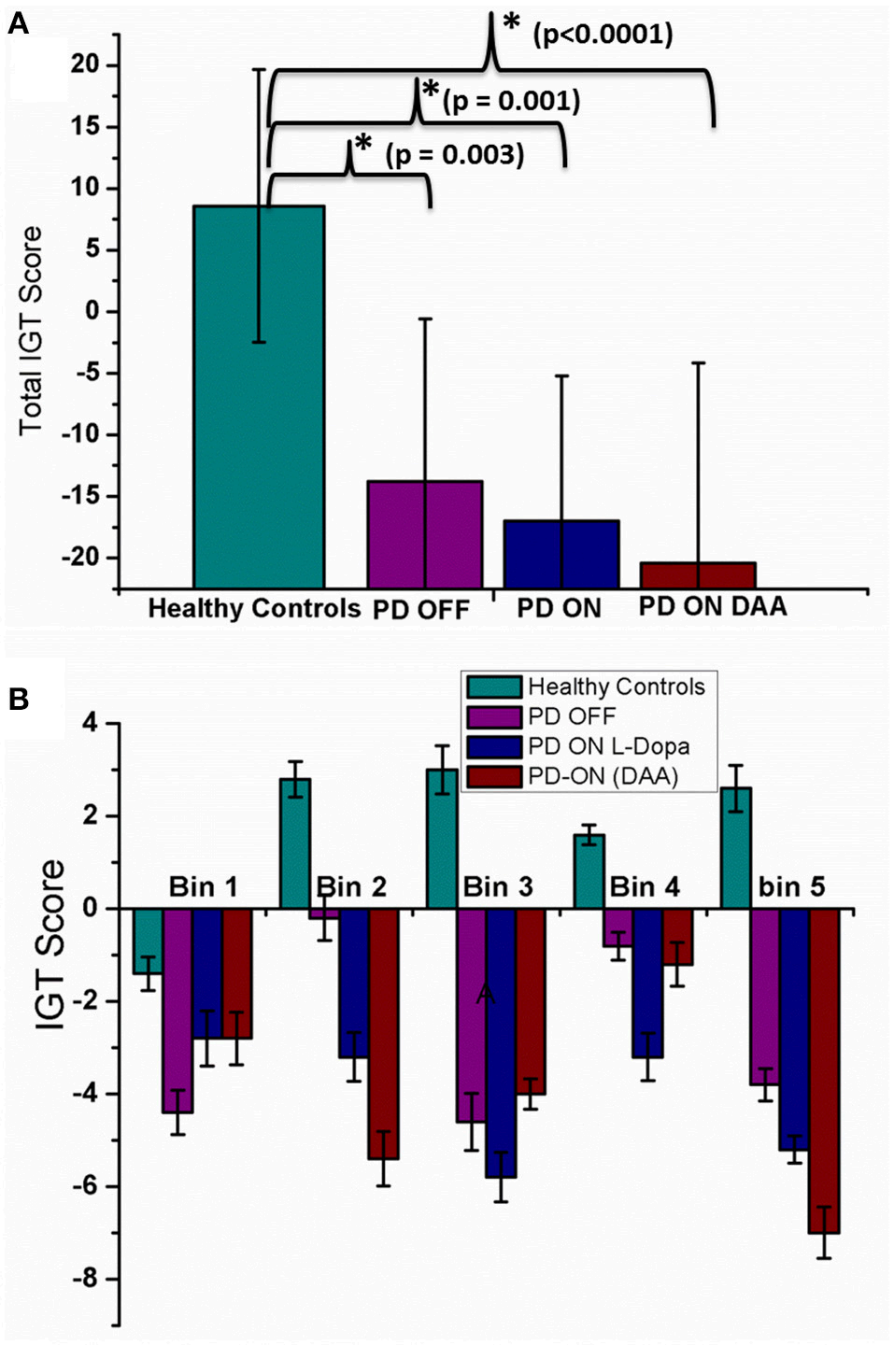
**Mean IGT score obtained from spiking model with standard error (SE) for four conditions, HC, PD OFF, PD ON (L-DOPA), and PD-ON (DAA) (A)** Total IGT score **(B)** bin score.

Total IGT scores for HC, untreated PD, medically treated PD (L-DOPA and DAA) were significantly different [*F*_(3, 36)_ = 9.813, *P* < 0.0001] [Figure 3A, Table A.V (Datasheet in Supplementary Material)]. *Post-hoc* analysis indicated a significant difference between HC and the other three conditions: untreated PD (*P* = 0.003), medically treated PD L-DOPA (*P* = 0.001) and DAA (*P* < 0.0001). No statistically significant difference was observed between the two medically treated PD (L-DOPA and DAA) conditions.

In HC condition, the score was negative in the 1st bin, but changed to positive at the 2nd bin and continued to increase, a trend that was absent in both untreated and medically treated PD (both L-DOPA and DAA) conditions (Figure 3B). The mean scores [Table A.V Datasheet in Supplementary Material)] for 1st bin of HC, untreated PD, medically treated PD L-DOPA and DAA were similar (*F* = 0.684, *P* = 0.568). For both 2nd and 3rd bins medically treated PD condition performed worse than healthy controls [bin 2 showed significant difference between HC, medically treated PD L-DOPA and medically treated PD DAA (*P* < 0.005); bin 3: The scores were significantly different between HC, medically treated PD L-DOPA (*P* = 0.01) and medically treated PD DAA, *P* = 0.025)]. Though untreated PD had lower scores compared to HC and higher scores compared to treated PD in both the bins, these differences did not reach statistical significance. The mean scores for 4th bin among the four conditions were not significantly different. For the 5th bin, except HC, all other conditions showed poor performance (*F* = 8.744, *P* < 0.0001). The individual bin scores obtained in each of the above described condition are given in Table 2.

**Table 2:** **The mean IGT scores with standard deviation for HC, untreated PD, medically treated PD (L-DOPA and DAA) at total and individual bin levels and total IGT score**.

**Condition**	**Score bin 1**	**Score bin 2**	**Score bin 3**	**Score bin 4**	**Score bin 5**	**Total score**
HC	−1.40±3.65	2.8±3.8	3.0±5.2	1.6±2.1	2.6±4.99	8.60±11.07
PD OFF	−4.4±4.78	−2±4.85	−4.6±6.2	−0.8±3.01	−3.8±3.45	−13.8±13.21
PD ON-LDOPA	−1.8±5.96	−2.8±5.26	−4.8±3.3	−3.2±5.1	−4.4±2.95	−17±11.78
PD ON-DAA	−2.8±5.67	−5.4±5.9	−2.6±5.9	−1.2±4.733	−7±5.5	−20.4±16.27

**Figure 4:**
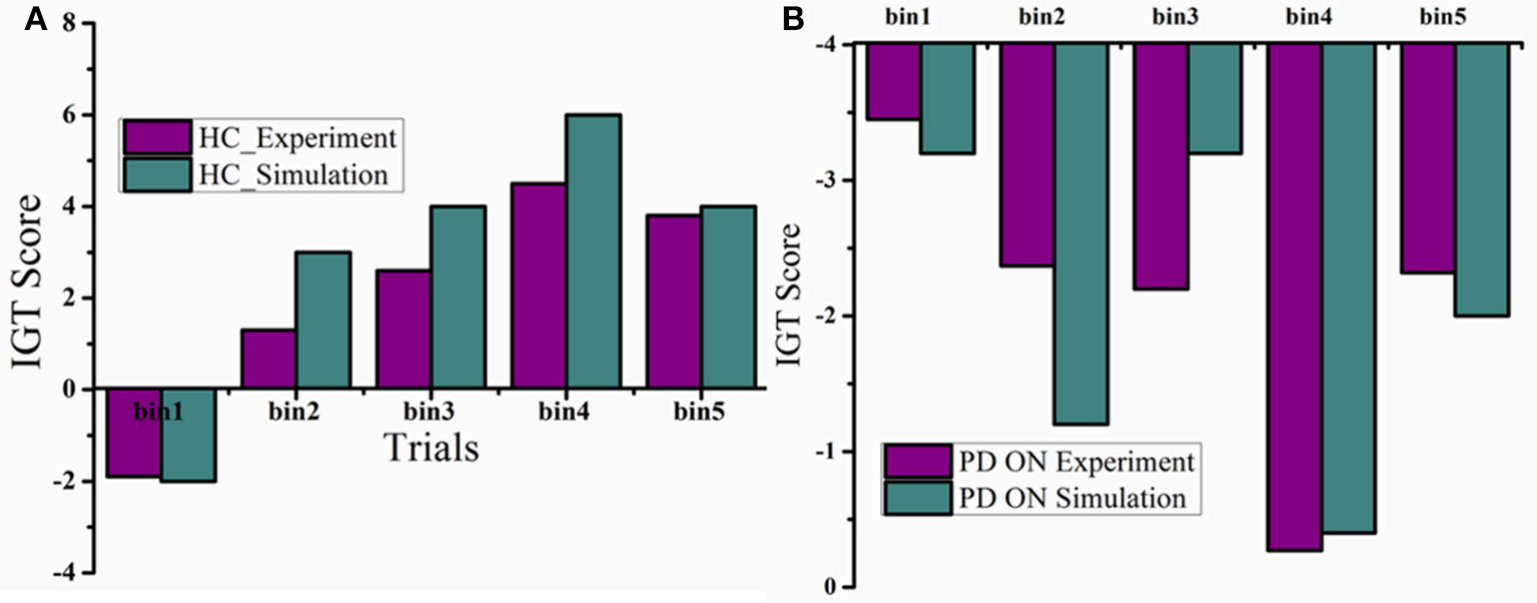
**IGT performance results were redrawn from Gescheidt et al. (2012). (A)** HC from experiment and simulation. **(B)** Medically treated PD patients from experiment and simulation (L-DOPA).

The mean IGT score values (Figure 4A) for HC, obtained from experiment (Gescheidt et al., 2012) and simulation (Figure 4B) were not significantly different (*P* = 0.19). Similarly the mean IGT score values obtained from medically treated PD subjects (Gescheidt et al., 2012) and simulation were not significantly different (*P* = 0.74).

### IGT-PD condition with and without stimulation

**Figure 5:**
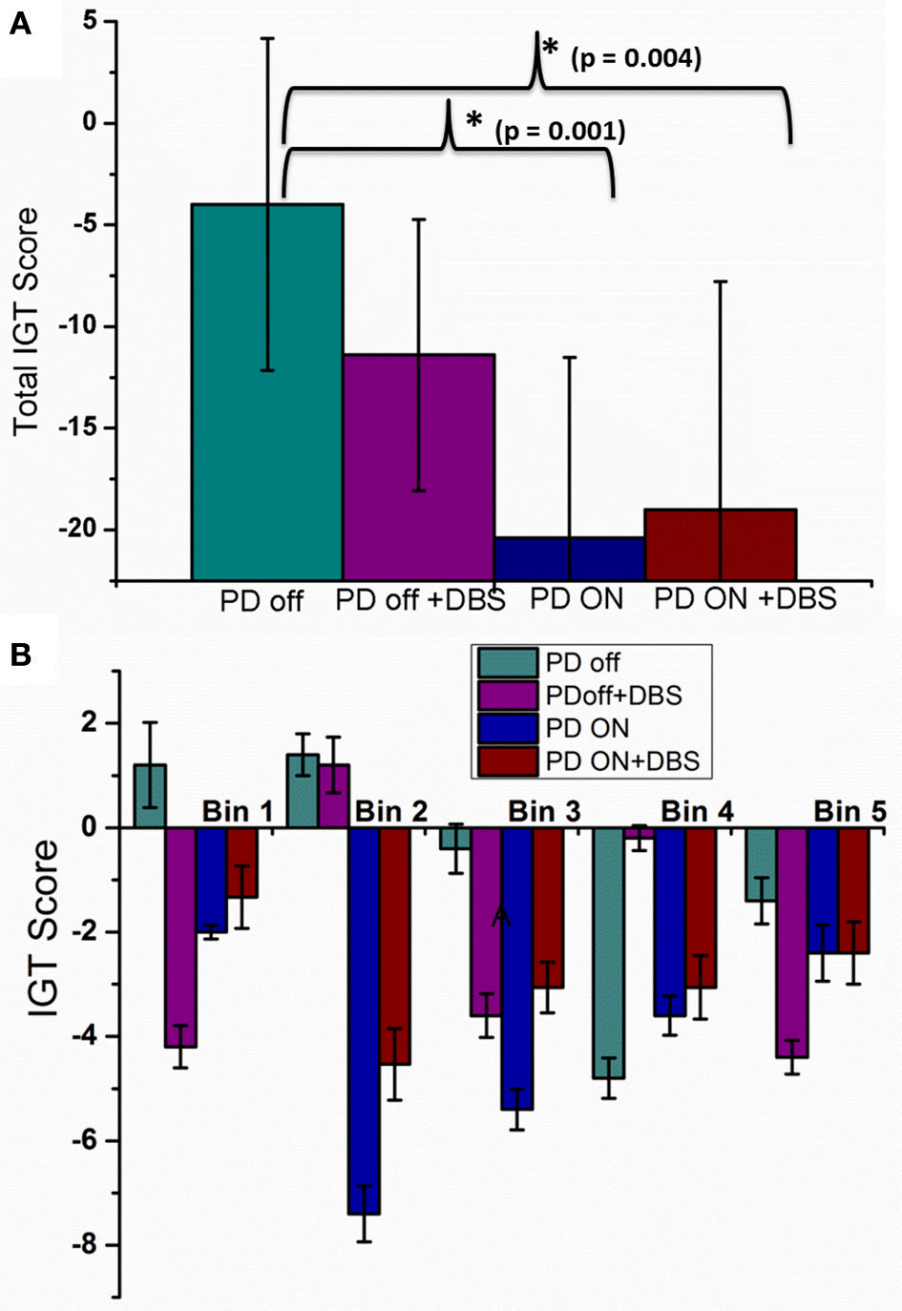
**The mean IGT score with SE obtained from spiking model calculated for four conditions; PD OFF, PD OFF+DBS, PD ON, PD ON+DBS (A)** Total IGT score and **(B)** bin score.

Total IGT score was negative for untreated PD, untreated PD with stimulation, medically treated PD and medically treated PD with stimulation (Figure 5A) with a significant difference among them [*F*_(3, 36)_ = 7.24, *P* = 0.001]. *Post-hoc* analysis revealed that medically treated PD (*P* = 0.001) and medically treated PD with stimulation (*P* = 0.004) had worse performance compared to untreated PD. No significant difference was observed between medically treated PD with and without stimulation.

A significant difference was observed among the 4 conditions (Figure 5B) for bin 1[*F*_(3, 36)_ = 3.24, *P* = 0.033] [Tables A.VI, A.VII (Datasheet in Supplementary Material)]. *Post-hoc* analysis indicated a significant difference only between untreated PD and untreated PD with stimulation (*P* = 0.039). For the 2nd bin [*F*_(3, 36)_ = 5.58, *P* = 0.003], medically treated PD performed worse compared to untreated PD (*P* < 0.008), no significant effect of stimulation was noted. No significant difference was observed for IGT score for the last three bins. The individual bin scores obtained in each of the above described condition are given in Table 3.

**Table 3:** **The mean IGT scores with standard deviation for untreated PD, medically treated PD (L-DOPA) with and without stimulation at total and individual bin levels**.

**Condition**	**Score bin 1**	**Score bin 2**	**Score bin 3**	**Score bin 4**	**Score bin 5**	**Total score**
PD OFF	1.2±4.02	1.4±4.7	−0.4±3.86	−4.8±4.4	−1.4±5.73	−4±8.17
PD OFF + stim	−4.2±4.05	1.2±5.3	−3.6±4.19	−0.2±2.3	−4.4±3.23	−11.4±6.67
PD ON	−2±1.33	−7.4±5.33	−5.4±3.89	−3.6±3.7	−2.4±5.4	−20.4±8.98
PD ON + stim	−3.4±5.96	−3.4±2.1	−4.2±4.8	−4.2±6.07	−2.9±6	−19±11.20

**Figure 6:**
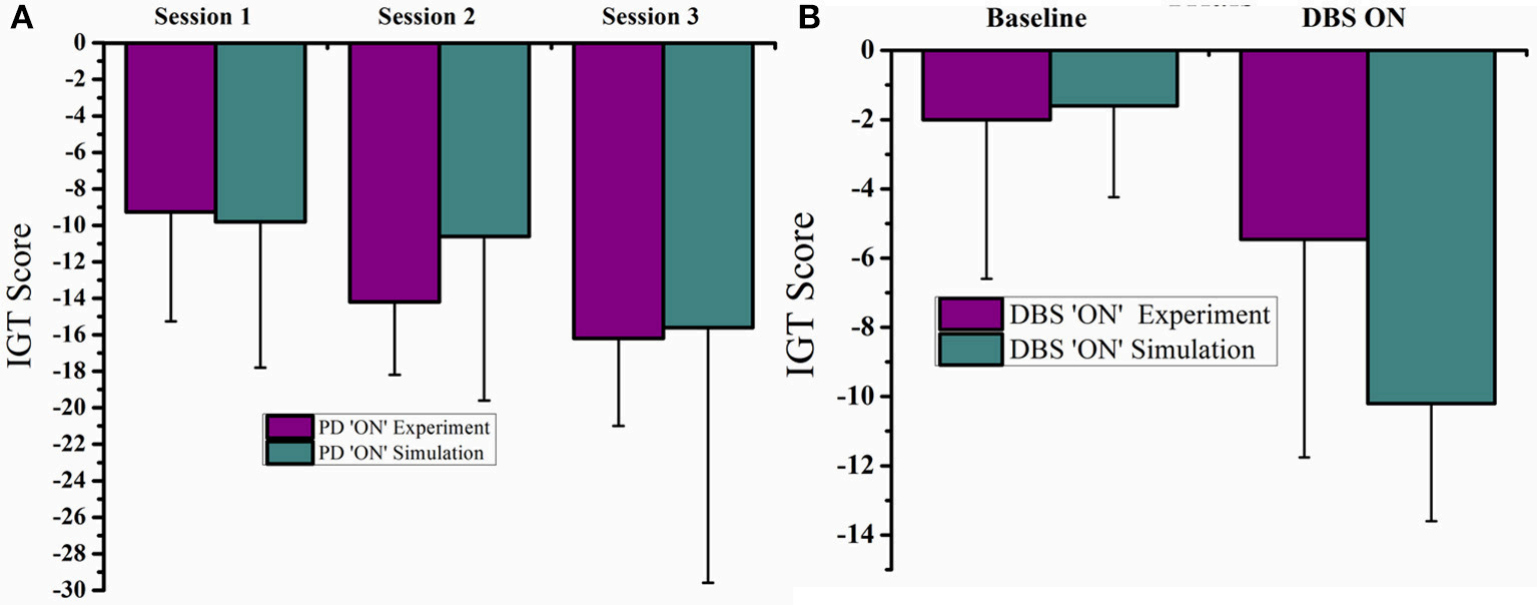
**Plots of IGT scores calculated for PD “ON” and PD ON+DBS condition**. The experimental results are redrawn from Oyama et al. (2011). **(A)** PD “ON” controls from experiment and simulation for 3 sessions **(B)** DBS subjects for baseline and DBS ON.

The mean IGT score values obtained from medically treated PD (PD “ON”) subjects from experiment (Oyama et al., 2011) and simulation (Figures 6A,B) were statistically similar (*P* = 0.42). Similarly the mean IGT score values for PD with STN-DBS (DBS “ON”) experiment and simulation were similar (*P* = 0.55).

### Effect of DBS electrode parameters on IGT score

**Figure 7:**
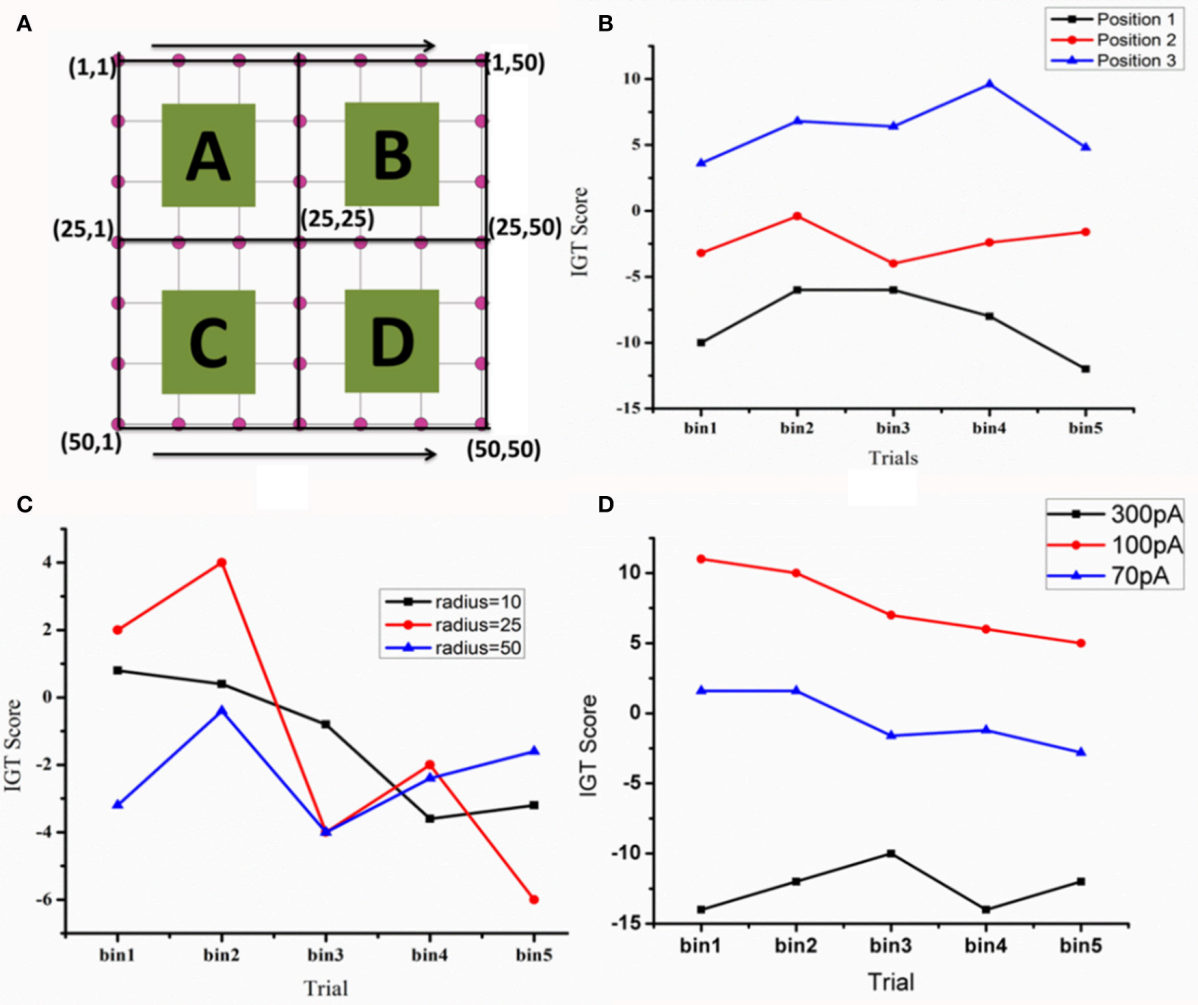
**The IGT score. (A)** Shows the STN network (=50 × 50) with quadrants that receive input from each of the corresponding decks **(A–D) (B)** The IGT score for three electrode positions [Position 1—in first quadrant with electrode center at lattice point (13,13), Position 2—center of the electrode at the lattice point (25,25), and Position 3- center of the electrode at the lattice point (38,38) in the fourth quadrant]. **(C)** For the electrode at position-2, the spread of the current (σ) was varied **(D)** the effect of DBS current amplitude (70, 100, and 300 pA) on IGT scores when the electrode is placed in position 3.

**Figure 8:**
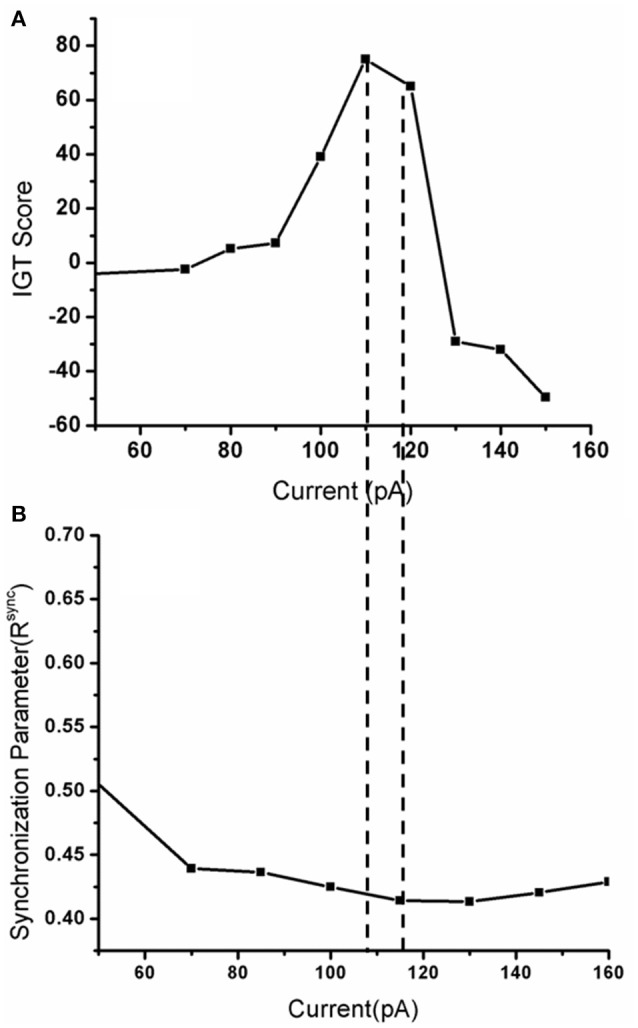
**Shows (A)** The IGT score obtained when the DBS current was increased from 0 (only medication) to up to 150 pA with frequency 130 Hz, biphasic mode. **(B)** The corresponding synchrony levels observed in STN neurons for that stimulation current.

When the electrode position (positions explained in Figure 7 legend) was changed such that stimulation (for untreated PD) is given selectively to a part of the STN module corresponding to each deck in IGT, we observed a significant variation in the IGT score (Figure 7B) (*P* < 0.0001).On changing the spread of DBS current (Figure 7C), there was a trend toward better performance with lower radius of spread (σ = 10), which, however, did not reach statistical significance (*P* = 0.67). We also observed a lower IGT score at higher (=300 pA) and lower (=70 pA) currents compared to that obtained from optimal current (=100 pA) (*P* < 0.001) (Figure 8A). Interestingly, the current range where the highest IGT score was obtained was nearly the same range where the synchrony in STN neurons was observed to be lowest (Figure 8B). The underlying cause for such an effect (Figures 7D, 8A,B), was investigated by observing the spiking activity of STN for both optimal (=100 pA) and high (=300 pA) current scenarios. At optimal current levels, DBS desynchronized the activity of the STN neurons (Figure 2F) that received the stimulation, leading to the selection of the stimulated panel (Figure 2D; Figure A1; purple line) and a positive IGT score. On the contrary, at non-optimal (=300 pA) current amplitudes, the corresponding STN neuron activity is increased leading to de-selection of the panel “D” (Figure A1).

## Discussion

We built a computational spiking basal ganglia network model to understand the effects of STN stimulation on impulsivity. We first tested the spiking network model, by comparing its simulated activity with the known pathophysiological alterations in PD. It has been observed that desynchronized, irregular STN activity observed in HC changes into synchronized bursting behavior in PD (Wilson and Bevan, 2011), which is also observed in STN neurons of MPTP treated primates (Bergman et al., 1994). This bursting oscillatory activity (Plenz and Kital, 1999) was also observed in our spiking model which correlated with tremor frequency, suggesting that the spiking model has the ability to reflect pathological STN activity.

### Effect of PD and dopaminergic therapy on IGT performance

Overall IGT performance was poor in untreated and medically treated PD conditions compared to HC. Medically treated PD condition did worse than HC and untreated PD, regardless of the type of medication used. Analysis of bin scores revealed that learning of the task was poor in all PD conditions compared to HC. Medically treated PD condition resulted in lower bin scores from the 2nd bin onwards, suggesting significantly impaired learning of the task, while in untreated PD; a significantly low bin score compared to HC was seen only in the last bin. The model in treated PD condition does not learn from its action outcomes (rewards/punishments) and wanders among the decks, which is reflected in the negative IGT score (Figure 3). This behavior is similar to that previously reported in a probabilistic action selection paradigm where PD “ON” subjects fail to avoid punitive choices (Frank et al., 2007). Physiologically this behavior is attributed to excess DA levels in striatum (Frank et al., 2007). In the model, striatal weights were positively updated even in punishment situation due to dopaminergic medication (δ_*med*_), leading to the selection of wrong choice. Clinical studies have identified dopamine agonists to be associated with higher risk of impulsivity, mediated through their D3 receptor affinity. We did not specifically model the D3 receptor activity and did not observe a higher risk with DAA (which selectively increased the D2 weight) compared to L-DOPA in our model.

### Effect of STN stimulation

Stimulation applied to the entire STN module did not result in a significant deterioration of overall IGT performance (Figure 5A). However, stimulation significantly impaired performance in the 1st bin when applied to untreated PD condition (Figure 5B). It is during these early trials that most of the learning regarding deck's reward pattern happens and this learnt information is used for future card selection. Stimulation seems to affect this learning ability, making the model performance worse as trials progress. Similar behavior is also reported in clinical experiments, where it was observed that PD patients with stimulation tend to overestimate their choices (Florin et al., 2013). No significant change was observed between medically treated PD with and without stimulation. This could be due to an overriding effect of dopaminergic medication over stimulation.

A few aspects of DBS that are specific to a PD patient who receives stimulation are the active contact point in the electrode, the amplitude of the current and the spread due to the current. Keeping this mind, we first changed the position of electrode within the STN nucleus (Figures 7A,B) and observed a significant change in IGT score. Physiologically, it might be possible that stimulation of different active points (which are 0.5–1 mm apart in the electrode) could lead to differential activation of neurons. This activation not only leads to overall difference in the current spread (McIntyre et al., 2004) but also in the activity of neurons that receive input from a different sources. This controlled activation might eventually changes the behavior (Witt et al., 2013).

Apart from position, another DBS parameter thought to significantly influence cognition is current amplitude. Various computational and experimental studies showed that the volume of tissue activated is dependent on the stimulating current amplitude (McIntyre et al., 2004; Arle et al., 2007; Yousif et al., 2010). We observed that a high stimulation current can increase the firing rate of STN neurons that received the stimulation whereas sufficiently low amplitude current would just desynchronize the activity. This change in STN, in terms of spiking activity during optimal and non-optimal (Figure A1; for a fixed position) stimulation currents might be the reason behind conflicting results observed at behavioral level (Figure 7D). For instance, de-selection of panel “D” increases probability of selection of other panels. In Figure A1, the selected panel was “A” (blue line) is a highly rewarding panel when viewed at a shorter time scale but punitive in long term. The model selects this panel due to its inability to learn the punishments associated with it. This selection of other panels (A and B are disadvantageous, and only C is rewarding) gave rise to a negative IGT score with high current.

With the above results one can consider the possibility that stimulation current when applied to the corresponding topographical areas of the panels within STN might lead to inhibition/facilitation of the corresponding panel selection depending on the current amplitude. To relate the above results physiologically, we suggest a role for the parallel functional loops within BG nuclei (Alexander and Crutcher, 1990) (motor, cognitive, and the limbic loops) and topographical mapping in impulsive behavior. A coarse functional organization is also observed within STN nucleus with motor and cognitive areas being adjacent to each other (Temel et al., 2005). We suspect that the highly variable cognitive outcomes in experimental studies could be correlated to electrode position and current spread (Figures 7B–D, 8A). These results are similar to those observed in the clinical study where a decrease in performance (hit rate) was observed when the position of the electrode was changed (Hershey et al., 2010).

Limitations of our model include the connectivity pattern within BG nuclei (GPe-GPi connection and hyper direct pathway is not included) and neuronal number. More elaborate modeling studies are required to further explore the effect of electrode position and stimulus waveform on motor and cognitive aspects of the PD patients. Although the human STN is known to be organized into motor and cognitive sub territories, a further topographical division into separate areas for various choices is yet unconfirmed. Nevertheless, our model reflects the pathophysiology of STN in PD and predicts behavioral changes similar to clinical data. Our results yield valuable information on the effect of electrode position and current amplitude on behavioral and cognitive outcomes of STN stimulation in PD that may help in the development of optimal stimulation protocols in a clinical setting.

## Author contributions

AM: Designed the study, built the computational model and performed the simulations, Manuscript Preparation and Analysis. VSC: Designed the study, built the computational model and performed the simulations, Manuscript Preparation and Analysis. RR: Designed the study, Manuscript Preparation and Analysis. AK: Designed the study, Manuscript Preparation and Analysis. SS: Analysis.

### Conflict of interest statement

The authors declare that the research was conducted in the absence of any commercial or financial relationships that could be construed as a potential conflict of interest.
