# Supplementary material for: Electrode Position and Current Amplitude Modulate Impulsivity after Subthalamic Stimulation in Parkinsons Disease—A Computational Study
Source: Front Physiol. 2016 Nov 29;7:585. doi: 10.3389/fphys.2016.00585 (PMC5126055; doi:10.3389/fphys.2016.00585)
Supplement: Supplementary file 1 [file DataSheet1.DOCX]

**Synaptic Connections in spiking BG model**

The synaptic connectivity between the nuclei was considered as one to one as in ([Dovzhenok and Rubchinsky, 2012](#_ENREF_25)) and was modeled as (similar to ([Humphries et al., 2009](#_ENREF_50)))

$\tau_{\mathrm{Re}cep}*\frac{dh_{ij}^{x\to y}}{dt}=-h_{ij}^{x\to y}(t)+S_{ij}^{x}(t)$ (A.1)

$I_{ij}^{x\to y}(t)=W_{x\to y}*h_{ij}^{x\to y}(t)*(E_{\mathrm{Re}cep}-V_{ij}^{y}(t))$ (A.2)

The effect of voltage-dependent magnesium channel on NMDA current ([Jahr and Stevens, 1990](#_ENREF_55)) was modeled as,

$B_{ij}(v)=\frac{1}{1+(\frac{Mg^{2+}}{3.57}*e^{-0.062*V_{ij}^{y}(t)})}$ (A.3)

Where τ_Recep_= decay constant for synaptic receptor, E_Recep_=receptor associated synaptic potential (Recep=AMPA/GABA/NMDA), S^x^_ij_=Spiking activity of neuron ‘x’ at time‘t’, h_ij_^x→y^= gating variablel for the synaptic current from ‘x’ to ‘y’,W_x→y_= synaptic weight from neuron ‘x’ to ‘y’, Mg^2+^ =Magnesium ion concentration and V^y^_ij_= membrane potential of the neuron ‘y’. The time constants of GABA, AMPA and NMDA in STN and GPe were chosen from ([Götz et al., 1997](#_ENREF_37)) are given in Table A.I. All the synaptic connections with their respective variables are described in Table A.II and the values of parameters are given in Table A.I.

**Lateral Connections in STN and GPe neurons**

$R_{s}=\frac{r_{s}}{(cD21*DA)};R_{g}=\frac{r_{g}}{(1-cD21*DA)};w_{ij,pq}^{m\to m}=A_{m}*e^{\frac{-d^{2}_{ij,pq}}{R_{m}^{2}}},d_{ij,pq}^{2}=(i-p)^{2}+(j-q)^{2}$ (A.4)

$I_{ij}^{\mathrm{Re}ceplat}=B_{ij}\left(v\right)*\sum_{p=1}^{n} \sum_{q=1}^{n} w_{ij,pq}^{x\to x}*h_{ij}^{\mathrm{Re}cep\to x}(t)*(E_{\mathrm{Re}cep}-V_{ij}^{x}(t))$ (A.5)

where r_s_ = constant variance of STN Gaussian, r_g_= constant variance of GPe Gaussian, R_s_ = changed variance of STN Gaussian due to the effect of dopamine, R_g_= changed variance of GPe Gaussian due to the effect of dopamine, cD21= a constant that determines the effect of DA on STN and GPe laterals, w_ij_^m→m^=lateral weight matrix of neuron‘m’at location (i, j), d_ij, pq_= distance from center neuron (p,q), R_m_=R_g_ (or) R_s_, A_m_=strength of lateral synapse, m =STN or GPe neuron. All parameter values are given in Table A.I.

$W_{x\to y}=\left(1-cd2*DA\right)*w_{x\to y}$ (A.6)

where the synapses are GPe→STN and STN→GPe. A similar method for DA-dependent synaptic modulation on striatal neurons was also used in ([Humphries et al., 2009](#_ENREF_50)).

Table A.I gives the values and the description of the parameters used in the model and simulation.

| **Parameter** | **Values with description** | | | |
| --- | --- | --- | --- | --- |
|  | STN | | GPe | GPi |
| Izhikevich parameters | a=0.005,b=0.265,c=-65, d=1.5 | | a=0.1,b=0.2,c=-65, d=2 | a=0.1, b=0.2, c=-65, d=2 |
| External current (I) | I_STN_=30 | | I_GPe_=10 | I_GPi_=10 |
| W_StrD1→GPi_ | 0.8 | | Synaptic weight between D1 striatum and GPi | |
| W_StrD2→GPe_ | 1 | | Synaptic weight between D2 striatum and GPe | |
| DA | 0.1 to 0.9 in increments of 0.1 | | | |
| A_D1_ | 10 | Amplitude of GABAergic current from D1 striatum to GPi neurons due to DA | | |
| A_D2_ | 7.5 | Amplitude of GABAergic current from D2 striatum to GPe neurons due to DA | | |
| λ^Str^ | 7.5 | Slope of the Gain functions (cD1 and cD2) | | |
| Mg^2+^ | 1nM | Concentration of Magnesium ions in nM | | |
| E_AMPA_ | 0mv | Synaptic potential of AMPA receptor-associated channel | | |
| E_NMDA_ | 0mV | Synaptic potential of NMDA receptor-associated channel | | |
| E_GABA_ | -60mV | Synaptic potential of GABA receptor-associated channel | | |
| w_sg_ | 1 | Synaptic weight for excitatory STN to GPe projection | | |
| w_gs_ | 20 | Synaptic weight for inhibitory GPe to STN projection | | |
| cd2 | 0.1 | Parameter that affects the STN→GPe (w_sg_) and GPe→STN(w_gs_) weights | | |
| τ_AMPA_ | 6ms | Time decay constant for AMPA receptor | | |
| τ_NMDA_ | 160ms | Time decay constant for NMDA receptor | | |
| τ_GABA_ | 4ms | Time decay constant for GABA receptor | | |
| τNMDA_GPi | 67ms | Time decay constant for NMDA receptor of GPi neurons | | |
| r_s_ | 1 | Radius of STN laterals Gaussian | | |
| r_g_ | 0.5 | Radius of GPe laterals Gaussian | | |
| cD21 | 0.1 | Parameter that affects the radius of STN and GPe laterals | | |
| A_GPe_ | 1 | Synaptic strength within GPe laterals | | |
| A_STN_ | 0.2 | Synaptic strength within STN laterals | | |
| nlatSTN_ | 5 | # of lateral connections considered in STN neurons | | |
| nlatGPe_ | 11 | # of lateral connections considered in GPe neurons | | |
| w_STN→GPi_ | 1.15 | Synaptic weight between STN and GPi | | |

Note: STN= Sub Thalamic Nucleus, GPe= Globus Pallidus Externa, GPi= Globus Pallidus Interna

**Total Synaptic currents received by each neuron**

Total synaptic currents received by GPe neurons

The total synaptic current received by a GPe neuron at lattice position (i,j) is the summation of GABAergic input from the D2-expressing striatal MSNs ([Gerfen et al., 1990](#_ENREF_33)) (eqn. A.5), glutamatergic current from STN considering both AMPA and NMDA currents ([Götz et al., 1997](#_ENREF_37)) (eqn. A.5) and the inhibitory lateral current form other GPe neurons (eqn. A.8). The influence of DA on the GABAergic current from D2 striatum to GPe neuron ([Hadipour-Niktarash et al., 2012](#_ENREF_42)) was accounted by the variable cD2.

$I_{ij}^{GPesyn}=I_{ij}^{GABAlat}+I_{ij}^{NMDA\to GPe}+I_{ij}^{AMPA\to GPe}+ I_{ij}^{StrD2\to GPe}*cD2$ (A.7)

$cD2=\frac{A_{D2}}{1+\exp(\lambda^{Str}*DA)}$ (A.8)

Where I_ij_^GABAlat^ = the inhibitory lateral GABAergic current from other GPe neurons, I_ij_^NMDA→GPe^ = excitatory glutamatergic current from STN neuron due to NMDA receptor, I_ij_^AMPA→GPe^ = excitatory glutamatergic current from STN neuron due to AMPA receptor, I_ij_^StrD2→GPe^ = inhibitory GABAergic current from D2 striatum, cD2= Gain parameter that affects the GABAergic D2 striatal current.

Total synaptic currents received by STN neurons

The total synaptic current received by an STN neuron at lattice position (i,j) is summation of GABAergic current from GPe neurons([Fan et al., 2012](#_ENREF_27)) (eqn. A.5) and glutamatergic input (both AMPA and NMDA) from other STN ([Kita et al., 1983](#_ENREF_58)) eqn.(A.8).

$I_{ij}^{STNsyn}=I_{ij}^{GABA\to STN}+I_{ij}^{NMDAlat}+I_{ij}^{AMPAlat}$ (A.9)

Where I_ij_^NMDAlat^ = excitatory glutamatergic current from collateral STN neurons due to NMDA receptor, I_ij_^AMPAlat^ = excitatory glutamatergic current from collateral STN neurons due to AMPA receptor,I_ij_^GABA→STN^ = inhibitory GABAergic current from GPe neuron.

Total synaptic currents received by GPi neurons

The total synaptic current received by a GPi neuron at lattice position (i,j) is a summation of GABAergic currents from D1 striatal neurons ([Gerfen et al., 1990](#_ENREF_33))and glutamatergic (both AMPA and NMDA) input from STN neurons ([Gerfen and Surmeier, 2011](#_ENREF_34)). The increase in GABAergic current from D1 striatum to GPi neurons due to DA modulation ([Kliem et al., 2007](#_ENREF_60)) was taken into account by the variable cD1.

$I_{ij}^{GPisyn}=I_{ij}^{NMDA\to GPi}+I_{ij}^{AMPA\to GPi}+I_{ij}^{StrD1\to GPi}*cD1$ (A.10)

$cD1=\frac{A_{D1}}{1+\exp(-\lambda^{Str}*(DA-1))}$ (A.11)

Where I_ij_^NMDA→GPi^ = excitatory glutamatergic current from STN neuron due to NMDA receptor, I_ij_^AMPA→GPi^ = excitatory glutamatergic current from STN neuron due to AMPA receptor, I_ij_^StrD1→GPi^ = inhibitory GABAergic current from D1 striatum, cD1= Gain parameter that affects the GABAergic striatal current.

Table A.II gives a description of all the synaptic variables of various synaptic currents modeled using eqns. (A.4, A.8) in Section 2.2.1

| **Variable** | **Description** |
| --- | --- |
| $h_{ij}^{StrD1x\to GPi }$ | Gating variables for GPi neuron due to GABAergic projections from D1 striatum. ‘x’ represents the input #. For example, if there are 2 inputs presented to the model, x=1, 2. |
| $h_{ij}^{StrD2x\to GPe }$ | Gating variable for GPe neuron due to GABAergic projections from D2 striatum |
| $h_{ij}^{AMPA\to GPe}/h_{ij}^{NMDA\to GPe}$ | Gating variable for GPe neuron due to glutamatergic input from STN due to either NMDA or AMPA receptor. |
| $h_{ij}^{GABA\to STN}$ | Gating variable for STN neuron due to GABAergic input from GPe neuron |
| $h_{ij}^{AMPA\to STN}/h_{ij}^{NMDA\to STN}$ | Gating variable for STN neuron due to glutamatergic input from its collaterals due to either NMDA or AMPA receptor. |
| $h_{ij}^{GABA\to GPe}$ | Gating variable for GPe neuron due to GABAergic input from its collaterals. |
| $I_{ij}^{StrD1\to GPi}$ | Inhibitory GABAergic current to GPi neuron from D1 striatum |
| $I_{ij}^{StrD2\to GPe}$ | Inhibitory GABAergic current to GPe neuron from D2 striatum |
| $I_{ij}^{AMPA\to GPe}/I_{ij}^{NMDA\to GPe}$ | Excitatory glutamatergic current (AMPA/NMDA) from STN neuron to GPe neuron |
| $I_{ij}^{GABA\to STN}$ | Inhibitory GABAergic current from GPe neuron to STN neuron |
| $I_{ij}^{AMPAlat}/I_{ij}^{NMDAlat}$ | Excitatory glutamatergic current (AMPA/NMDA) from STN neuron to STN neuron due to collateral synapses. |
| $I_{ij}^{GABAlat}$ | Inhibitory GABAergic current from GPe neuron to GPe neuron due to collateral synapses. |
| $S_{ij}^{GPe}$ | Spiking activity of GPe neuron at location (i,j) at time ‘t’. |
| $S_{ij}^{STN}$ | Spiking activity of STN neuron at location (i,j) at time ‘t’. |
| $S_{ij}^{Dxy}$ | Spiking activity of striatum at location (i,j) at time ‘t’. The variable ‘y’ represents either D1 striatum (=1) or D2 striatum (=2) for an input stimulus ‘x’. |

Note: STN= Sub Thalamic Nucleus, GPe= Globus Pallidus Externa, GPi= Globus Pallidus Interna

**Parameter values used in the model for each of the simulated 4 conditions**

**Case 1: STN activity in Parkinsonian state**

To test the effect of stimulation on STN activity, we added the stimulation current which was modeled as Gaussian distribution (eqn. A.16) to STN module. Fig. 2(c) shows an example plot of the DBS current in biphasic mode with a positive and negative peak. The pulse duration of the current was set to 200μs at a pulse frequency of 130Hz. The spread of current in STN is shown in the zoomed panel (Fig. 2c) where 1^st^ half of the biphasic pulse has a positive peak and 2^nd^ half is negative, distributed across the lattice( =50x50)

**Case 2: HC Vs PD OFF Vs PD ON**

To simulate the HC condition, the values of Cd1str (=15) and Cd2str (=7) (eqn. A.8 & A.11) were chosen. The synaptic weight between STN &GPi: w_STN→GPi_ (=1.5), D1 striatum to GPi: w_Str→GPi_(=4), D2striatum to GPe: w_Str→GPe_(=1) were used in the network to resemble physiological conditions where the activity of direct pathway (w_Str→GPi_) is higher than indirect pathway (w_Str→GPe_). The condition of PD was obtained by (a) clamping the value of delta (δ) (eqn. A.9) to negative value (δ_lim_= -150) (eqn. A.12). One way to simulate highly active indirect pathway in PD condition, is to increase the value of Cd2str (input from D2 striatum to GPe) from 7 to 25 [[61](#_ENREF_61), [62](#_ENREF_62)]. Similarly, increased excitatory input from STN to GPi weight (w_STN→GPi_) is simulated by increasing the synaptic weight from 1.5 to a value of 2.5 [[62](#_ENREF_62)]. The 2 medication conditions of PD were obtained by adding medication term (δ_med_=180) (eqn. A.12) to the clamped delta (δ_lim_), a way of simulating DA levels (due to medication) [[55](#_ENREF_55), [56](#_ENREF_56)]. To simulate the results similar to that in Geischeidt et al (2012), medication (δ_med_=20) was used.

**Case 3: Stimulation to STN neurons**

All the parameter values in the model are kept same as in case 2, except for the clamping value of delta (δ_lim_= -75). This was done because the experimental results that are to be compared with the simulation results were obtained from a different class of PD patients. To model the effect of medication (L-Dopa) the constant value of (δ_med_=120) was added to the clamped delta. Stimulation was given to STN and was modeled as biphasic pulses with pulse width (200μs), amplitude (100pA) and frequency (130Hz). The tip of electrode was assumed to be located at the center of STN module and the spread of current is assumed to follow Gaussian function with a radius (= 10) (eqn. A.14). The second set of results in this section relate to the study of the effect of DBS stimulation on IGT task performance compared with the study by Oyama et al (2011), where IGT task performance between PD ‘ON’ controls and ‘STN DBS’ patients was compared for 2 consecutive sessions (of 100 trials each). For the DBS group, the first session was baseline without DBS; the second session was with DBS ON. Oyama et al (2011) reported an increase in the total IGT score in the third session (DBS OFF) in DBS subjects, though no concrete explanation was provided regarding how such a response was obtained. The medication value was changed (δ_med_ =10) to replicate the experimental results.

**Case 4: Effect of DBS electrode parameters on IGT**

To check the effect of electrode position on IGT performance score, we changed the position of the electrode (Position 1- Quadrant 1with the center of the electrode at the lattice point (13, 13), Position 2- Center of the electrode at lattice point (25, 25), Position 3- In Quadrant 4 at lattice point (38, 38)) within the STN nucleus and ran the simulation for a total of 100 trials and calculated the IGT score for 5 bins. The frequency of the stimulation current was kept constant (=130 Hz) with amplitude (=100pA) in biphasic mode. The spread of the current was assumed to follow Gaussian distribution [[60](#_ENREF_60)] controlled by radius (σ=25). The current spread was limited to fixed number of neighboring neurons (=25).

**Synchronization parameter (R^sync^)**

To quantify the level of synchrony in the STN-GPe standalone network, we used the synchrony measure as defined in [[48](#_ENREF_48)].

The phase of j^th^ neuron is calculated as follows,

$\emptyset_{j}\left(t\right)=2*\pi*\frac{\left(T_{j,k}-t_{j,k}\right)}{(t_{j,k+1}-t_{j,k})}$ (A.12)$R^{sync}\left(t\right)*e^{i\theta\left(t\right)}=\frac{1}{N}\sum_{j=1}^{N} \text{e}^{\text{i}\emptyset_{j}\left(t\right)}$ (A.13)

where t_j,k_ and t_j,k+1_ are the onset times of k^th^ and k+1^th^ spike of the j^th^ neuron $T_{j,k}\in\left[t_{j,k},t_{j,k+1}\right]$, $\emptyset_{j}\left(t\right)$= phase of j^th^ neuron at time ‘t’, R^sync^ is the synchronization measure 0≤R^sync^≤1,$\theta$=Average phase of neurons, N=total number of neurons in the network.

**Reward and Penalty values in Iowa Gambling Task**

Table A.III indicates the reward, loss distribution from each of the decks.

|  | **Deck A** | **Deck B** | **Deck C** | **Deck D** |
| --- | --- | --- | --- | --- |
| **Gain per card($) with probability (=1)** | 100 | 100 | 50 | 50 |
| **Loss distribution($) for 10 cards** | [0,0,0,0,0,-150, -200,-250,-300,-350 | [0, 0, 0, 0, 0,0,0, 0, 0, -1250] | [0,0,0,0,0,-50,-50,-50,-50,-50] | [0, 0, 0, 0, 0, 0, 0, 0,-250] |
| **Probability of Loss** | 0.5 | 0.1 | 0.5 | 0.1 |
| **Net per 10 cards ($)** | -250 | -250 | 250 | 250 |

Table A.IV lists the Acronyms and parameters used in this article.

| **Variable/Acronym** | **Full form** |
| --- | --- |
| STN | Sub Thalamic Nucleus |
| PD | Parkinson’s Disease |
| DBS | Deep Brain Stimulation |
| IGT | Iowa Gambling Task |
| BG | Basal Ganglia |
| RL | Reinforcement Learning |
| DA | Dopamine |
| L-DOPA | Leva Dopa |
| DAA | Dopamine Agonist |
| GPe | Globus Pallidus externus |
| GPi | Globus Pallidus internus |
| Untreated PD/ PD OFF | PD condition without medication |
| Medically treated PD/ PD ON | PD condition with medication |
| HC | Healthy controls |
| MPTP | 1-methyl-4-phenyl-1,2,3,6-tetrahydropyridine |
| ICD | Impulse Control disorder |
| GABA | Γ- Aminobutyric acid |
| δ | Error term similar to dopamine |
| σ | Parameter that controls the spread of the current |
| $w_{i,k}^{D1}$ | Cortico-striatal weight to D1 striatum |
| $w_{i,k}^{D2}$ | Cortico-striatal weight to D2 striatum |
| A_DBS_ | Parameter that controls the amplitude of the DBS current |
| δ_lim_ | Clamped DA value resembling PD condition |
| δ_med_ | DA medication |
| η | (=0.1) Learning rate of the model |
| R^sync^ | Synchronization measure |

**Results**

Table A.V shows the mean IGT scores with standard deviation for HC, untreated PD, medically treated PD (L-DOPA & DAA) at total and individual bin levels and total IGT score.

| **Condition** | **Score bin 1** | **Score bin 2** | **Score bin 3** | **Score bin 4** | **Score bin 5** | **Total score** |
| --- | --- | --- | --- | --- | --- | --- |
| HC | -1.40±3.65 | 2.8±3.8 | 3.0±5.2 | 1.6±2.1 | 2.6±4.99 | 8.60±11.07 |
| PD OFF | -4.4±4.78 | -2±4.85 | -4.6±6.2 | -0.8±3.01 | -3.8±3.45 | -13.8±13.21 |
| PD ON-LDOPA | -1.8±5.96 | -2.8±5.26 | -4.8±3.3 | -3.2±5.1 | -4.4±2.95 | -17±11.78 |
| PD ON-DAA | -2.8±5.67 | -5.4±5.9 | -2.6±5.9 | -1.2±4.733 | -7±5.5 | -20.4±16.27 |

Table A.VI shows the mean IGT scores with standard deviation for untreated PD, medically treated PD (L-DOPA) with and without stimulation at total and individual bin levels

| **Condition** | **Score bin 1** | **Score bin 2** | **Score bin 3** | **Score bin 4** | **Score bin 5** | **Total score** |
| --- | --- | --- | --- | --- | --- | --- |
| PD OFF | 1.2±4.02 | 1.4±4.7 | -0.4±3.86 | -4.8±4.4 | -1.4±5.73 | -4±8.17 |
| PD OFF + stim | -4.2±4.05 | 1.2±5.3 | -3.6±4.19 | -.2±2.3 | -4.4±3.23 | -11.4± 6.67 |
| PD ON | -2±1.33 | -7.4±5.33 | -5.4±3.89 | -3.6±3.7 | -2.4±5.4 | -20.4 ± 8.98 |
| PD ON + stim | -3.4±5.96 | -3.4±2.1 | -4.2±4.8 | -4.2±6.07 | -2.9±6 | -19 ±11.20 |

The comparison of IGT score between untreated and treated PD with and without stimulation at bin level when the stimulation was given to the entire STN module.

Table A.VII shows the comparison between IGT score for each bin

| Bin | F(3,36) | P value |
| --- | --- | --- |
| 1 | 3.240 | 0.033 |
| 2 | 5.575 | 0.003 |
| 3 | 2.562 | 0.07 |
| 4 | 2.22 | 0.102 |
| 5 | 0.575 | 0.635 |

**
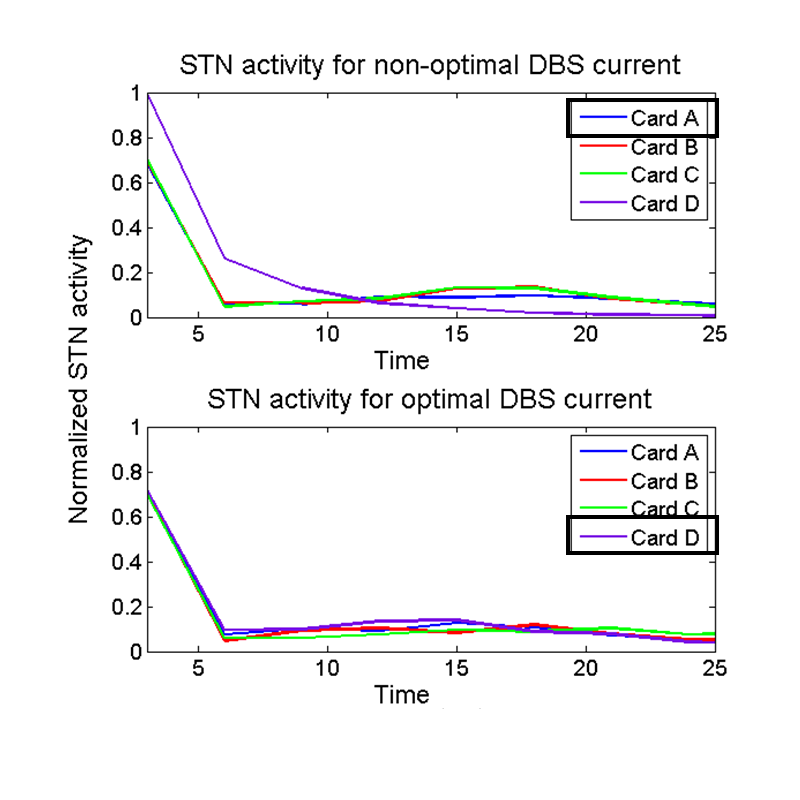
**

Figure A1 shows the normalized STN activity in each of the four quadrants (the sum of spikes of STN neurons corresponding to a quadrant divided with the highest of spikes obtained from the whole STN) when DBS current was provided at (a) non-optimal (=300pA) and (b) optimal (=100pA) amplitudes at position 3 (quadrant 4). The selected card in that trial is highlighted in the two cases respectively.
